# Recent Approaches for Cleaving the C─C Bond During Ethanol Electro‐Oxidation Reaction

**DOI:** 10.1002/advs.202308958

**Published:** 2024-02-11

**Authors:** Chenjia Liang, Ruiyao Zhao, Teng Chen, Yi Luo, Jianqiang Hu, Ping Qi, Weiping Ding

**Affiliations:** ^1^ School of Chemistry and Chemical Engineering Nanjing University Nanjing Jiangsu 210023 China; ^2^ Department of Aviation Oil and Material Air Force Logistics Academy Xuzhou Jiangsu 221000 China

**Keywords:** C1 path selectivity, C─C cleaving, CO_2_ selectivity, electrocatalyst, ethanol oxidation reaction

## Abstract

Direct ethanol fuel cells (DEFCs) play an indispensable role in the cyclic utilization of carbon resources due to its high volumetric energy density, high efficiency, and environmental benign character. However, owing to the chemically stable carbon‐carbon (C─C) bond of ethanol, its incomplete electrooxidation at the anode severely inhibits the energy and power density output of DEFCs. The efficiency of C─C bond cleaving on the state‐of‐the‐art Pt or Pd catalysts is reported as low as 7.5%. Recently, tremendous efforts are devoted to this field, and some effective strategies are put forward to facilitate the cleavage of the C─C bond. It is the right time to summarize the major breakthroughs in ethanol electrooxidation reaction. In this review, some optimization strategies including constructing core–shell nanostructure with alloying effect, doping other metal atoms in Pt and Pd catalysts, engineering composite catalyst with interface synergism, introducing cascade catalytic sites, and so on, are systematically summarized. In addition, the catalytic mechanism as well as the correlations between the catalyst structure and catalytic efficiency are further discussed. Finally, the prevailing limitations and feasible improvement directions for ethanol electrooxidation are proposed.

## Graphical abstract

The major breakthroughs of C─C bond cleavage strategies in ethanol electrooxidation reaction including construction of core–shell nanostructure with alloying effect, doping other metal atoms in Pt and Pd catalysts, engineering composite catalyst with interface synergism, and introducing cascade catalytic sites are systematically summarized. In addition, the prevailing limitations and feasible improvement directions for ethanol electrooxidation are proposed.

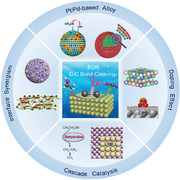

## 1 Introduction

Fuel cells have risen to prominence as an indispensable and prospective technology, particularly in the realm of heavy‐duty vehicles, in stark contrast to conventional thermal engines, renowned for their conversion of thermal energy sourced from fossil fuels into mechanical work.^[^ ^1^, ^2^ ^]^ These devices manifest an extraordinary capacity to directly transmute the chemical potential harbored within a diverse array of fuels (encompassing hydrogen, methanol, ethanol, and so on) into electrical energy.^[^ ^3^, ^4^, ^5^ ^]^ The energy‐conversion approach not only displays exceptional energy conversion efficiency but also proffers free pollution, on account of non‐limitation imposed by Carnot cycle, which traditionally governs thermal engines.^[^ ^6^, ^7^, ^8^ ^]^ Among various types of fuel cell, direct ethanol fuel cells (DEFCs) have evoked profound interest and attention in the scientific and industrial echelons, prominently featuring an elevated power density, a simple device system, and mild operating conditions.^[^ ^9^, ^10^ ^]^ Besides, as an environmentally‐friendly, renewable, non‐toxic, and portable fuel ethanol, its volumetric energy density (6.28 kWh·L^–1^) is much higher than those of methanol (4.05 kWh·L^−1^) and hydrogen gas (0.18 kWh·L^−1^, compressed at 20 MPa). The above characteristics make it possible for DEFCs to replace fossil fuel consuming internal combustion engines, especially in application scenarios such as heavy‐duty trucks and ocean freighter.

**Figure 1:**
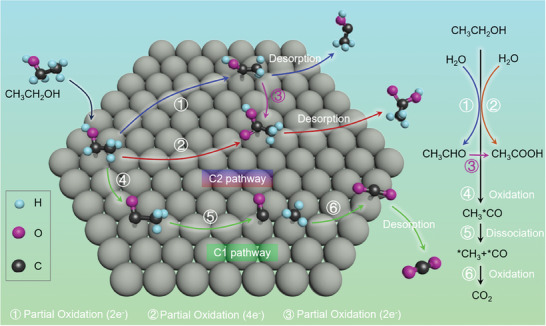
The schematic diagram of C1 and C2 pathways of EOR.

Up to now, platinum (Pt) and palladium (Pd) are still the state‐of‐the‐art anodic electrocatalysts in DEFCs.^[^ ^11^, ^12^ ^]^ However, pure Pt and Pd catalysts with high cost show the sluggish reaction kinetic for ethanol oxidation reaction (EOR) because of their unfulfilling C─C bond breaking capability and poor *CO intermediate tolerance, which seriously hinder the output of energy and power density for DEFCs.^[^ ^13^, ^14^ ^]^ Generally, two parallel and competing pathways are recognized for EOR, that are, the complete oxidation pathway (C1 pathway) and incomplete oxidation pathway (C2 pathway). The C2 pathway involves 2/4 electrons transferring without C─C bond breaking, resulting in the acetaldehyde/acetic acid as the final products (**Figure** **1**). Inversely, the preferred C1 pathway involves 12 electrons transferring with C─C bond breaking and producing CO_2_.^[^ ^15^ ^]^ Due to the high activation energy (87.3 kcal mol^−1^) of C─C bond breaking, the C1 selectivity of Pt and Pd catalysts is very low (<7.5%).^[^ ^15^, ^16^ ^]^ In addition, some EOR intermediates (such as *CO) can strongly adsorb on and occupy the active sites of catalysts, degrading the EOR performance significantly.^[^ ^17^, ^18^, ^19^, ^20^, ^21^ ^]^

Recently, some strategies have been put forward to improve the catalytic performance of Pt and Pd catalysts, such as alloying Pt and Pd with other oxophilic metals.^[^ ^20^, ^21^, ^22^, ^23^, ^24^ ^]^ The enhanced catalytic performance can be mainly attributed to the bifunctional mechanism. On the one hand, oxophilic metals can increase the lattice strain and modulate the electronic structure of Pt and Pd catalysts. On the other hand, the formed OH_ads_ (adsorbed hydroxyl) on the surface of oxophilic metals promote the oxidation of *CO. As a result, the C1 pathway selectivity on Pt/Pd alloy can be improved up to nearly 100% in alkaline electrolyte.^[^ ^22^, ^23^, ^24^, ^25^ ^]^ However, due to the leaching and dissolution of oxophilic metals in acid electrolyte, the stability of Pt and Pd catalysts should be further improved. Fortunately, a large number of research works have upgraded the electrocatalytic active centers and revealed the key processes of ethanol oxidation, which provide an important foundation for the development of EOR catalysts with the ability of C─C bonds cleaving.^[^ ^24^, ^25^, ^26^, ^27^, ^28^, ^29^ ^]^

This article reviews a series of efficient strategies for breaking C─C bonds during ethanol electro‐oxidation reaction, including constructing core–shell nanostructure with alloying effect, doping other metal atoms in Pt and Pd catalysts, engineering composite catalyst with interface synergism and introducing cascade catalytic sites in recent research (**Table** **1**). The ethanol electro‐oxidation mechanism on the catalysts mentioned is are further discussed. Finally, we undertake a comprehensive elucidation of the prevailing limitations of cleaving C─C bonds in the EOR process and provide some preliminary insights on feasible improvement directions to promote the industrialization development of DEFCs.

**Table 1:** Summary of the EOR catalysts, catalysis abilities, and design strategies.

Strategy	Catalysts	Condition	Special activity	Faraday efficiency of C1 pathway	Ref.
Pt‐/Pd‐ based Alloy	Pd‐Au HNS/C	1 M KOH + 1 M C_2_H_5_OH	11.5 mA cm^−2^	33.2%	[30]
	Pt_69_Ni_16_Rh_15_ NWs/C	0.1 M HClO_4_ + 0.5 M C_2_H_5_OH	2.49 mA cm^−2^	—	[32]
	FFT Pt‐Ir NRs	1 M KOH + 1 M C_2_H_5_OH	10.22 A cm^−2^	61.21%	[35]
	Pt_3_Ga/C	0.1 M HClO_4_ + 0.1 M C_2_H_5_OH	2.46 mA cm^–2^	—	[36]
	Pt_0.68_Cu_0.18_Ru_0.14_ NFs	0.1 M HClO_4_ + 0.5 M C_2_H_5_OH	7.90 mA cm^−2^	—	[37]
	Pd_8_Sb_3_ HPs/C	0.5 M NaOH + 0.5 M C_2_H_5_OH	29.30 mA cm^−2^	—	[38]
	Pd_50_W_27_Nb_23_/C	1 M KOH + 1 M C_2_H_5_OH	15.6 A mg_Pd_^−1^	55.5%	[39]
	PdCu SMPs	1 M KOH + 1 M C_2_H_5_OH	6.09 A mg^−1^_Pd_	72.1%	[40]
	a‐PdCu	1 M KOH + 1 M C_2_H_5_OH	15.25 A mg^−1^_Pd_	69.6%	[41]
	HEIs	0.1 M HClO_4_ + 0.2 M C_2_H_5_OH	47.50 mW cm^−2^	/	[45]
	Au@FePd‐0.5	1 M KOH + 1 M C_2_H_5_OH	20.2 mA cm^−2^	—	[49]
	Au@PtIr/C	1 M KOH + 1 M C_2_H_5_OH	8.3 A mg^−1^_AuPtIr_	57%	[50]
	PtCo/Co‐N‐C	0.1 M HClO_4_ + 0.5 M C_2_H_5_OH	3.95 A mg_Pt_^−1^	—	[53]
	Pt_3_Co@Pt/PC	0.1 M HClO_4_ + 0.1 M C_2_H_5_OH	0.79 mA µg_P_^t‐1^	—	[56]
	PtPb@PtIr_1_ HNPs/C	0.5 m H_2_SO_4_ + 1 m C_2_H_5_OH	7.53 mA cm^−2^	57.93%	[57]
	Pt‐Cu‐Rh HNs/C	0.1 m HClO_4_ + 0.5 m C_2_H_5_OH	7.3 mA cm^−2^	—	[66]
	PdZn/NC@ZnO	1 m KOH + 1 m C_2_H_5_OH	18.14 A mg_Pd_^−1^	—	[69]
	YO*x*/MoO*x*–Pt NWs	0.1 m HClO_4_ + 0.5 m C_2_H_5_OH	3.35 mA cm^−2^	—	[70]
	Rh–O–Pt	0.1 m HClO_4_ + 0.5 m C_2_H_5_OH	7.43 mA cm^−2^	—	[71]
Cascade electrocatalysis	Pt/Al_2_O_3_@TiAl	0.1 m HClO_4_ + 0.5 m C_2_H_5_OH	3.83 mA cm^−2^_Pt_	100%	[72]
Interface synergism	Rh–SnO_2_	0.1 m HClO_4_ + 0.5 M C_2_H_5_OH	213.2 mA mg_Rh_^−1^	72.8%	[17]
Doping effect	Ga–O–Pt_3_Mn	0.1 m HClO_4_ + 0.5 m C_2_H_5_OH	4.71 mA cm^−2^	—	[88]
	Rh_at_O–Pt NCs/C	0.1 m HClO_4_ + 0.2 m C_2_H_5_OH	≈0.9 mA cm^−2^	—	[97]
	a‐PdCu	1 m KOH + 1 m C_2_H_5_OH	15.25 A mg^−1^_Pd_	69.6%	[41]
	S_3.67_‐PtCu IM/C	1 m KOH + 1 m C_2_H_5_OH	20.42 mA cm^−2^	93.5%	[98]

## 2 Strategies to Improve the C1 Selectivity

### 2.1 Pt‐/Pd‐Based Alloys

Due to the substantial energy expenditure associated with the cleavage of the C─C bond, the C1 path selectivity in the EOR process remains constrained to a value below 7.5% when employing a solitary noble‐metal center, such as platinum (Pt) or palladium (Pd), as an electrocatalyst.^[^ ^30^ ^]^ Among them, only the Pt‐based center has EOR reaction activity under acidic conditions. One plausible approach to improve the selectivity efficiency of the C1 pathway is the conceptualization of Pt‐group alloys featuring diverse topological arrangements, which can manifest lattice strain effects, modulate active electronic structures, and expose a greater number of active centers.^[^ ^31^, ^32^, ^33^ ^]^

**Figure 2:**
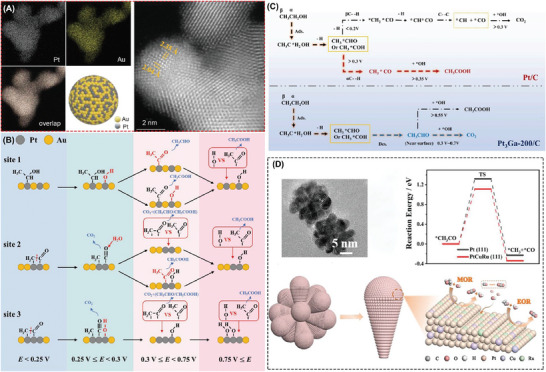
A) HAADF‐STEM images and elemental mapping of Pt_1_Au_1_/C. B) The schematic diagram of the ethanol electro‐oxidation mechanism with possible sites on the surface of Pt_1_Au_1_ in different potential ranges. Reproduced with permission.^[^ ^34^ ^]^ Copyright 2023, Springer. C) Proposed pathway of direct complete oxidation of ethanol through C2 intermediate on Pt_3_Ga/C, compared to traditional way on Pt/C. Reproduced with permission.^[^ ^36^ ^]^ Copyright 2023, American Chemical Society. D) HRTEM images of PtCuRu NFs and DFT‐calculated reaction mechanisms of C─C bond breaking on PtCuRu (111). Reproduced with permission.^[^ ^37^ ^]^ Copyright 2022, Wiley‐VCH.

As shown in **Figure** **2**A,B, the Sun group comprehensively elucidated the transformations occurring during ethanol electro‐oxidation across an in situ spectrum of active sites present on the PtAuRu alloy surface within distinct potential regimes.^[^ ^34^ ^]^ These insights carry substantial significance in guiding the strategic development of alloy catalysts characterized by enhanced C1 selectivity. Specifically, under low potentials (≤0.25 V), it was discerned that the continuous presence of two or three Pt atoms constituted the pivotal active centers responsible for cleaving the C─C bond, particularly when ethanol intermediate species were adsorbed in a bridged configuration. This adsorption configuration left carbon monoxide (*CO) and *CH*_x_* fragments preferentially adsorbed on the Pt sites and further oxidized to CO_2_. Moreover, Zhang et al reported notably heightened mass activity (4.18 A mg_Pt_^−1^) and superior C1 pathway selectivity (61.21%) in the case of 1D Pt–Ir alloy nanorods with tensile‐strained (100) facets as compared to the commercial Pt black catalyst.^[^ ^35^ ^]^

In more intricate acidic environments, an electrocatalyst denoted as Pt_3_Ga/C, as reported by Huang et al,^[^ ^36^ ^]^ had been employed for EOR. This catalyst exhibits a homogeneously distributed gallium (Ga) configuration on the surface of Pt nanoparticles; thus, ensuring an effective and infrequent generation of CO_2_ at a moderate potential (≈0.3 V vs SCE). This achievement is attributed to the catalyst's capacity for the direct and sustainable oxidation of the C2 intermediate, namely, acetaldehyde, as depicted in Figure 2C. The atomic spacing structure between platinum (Pt) and gallium (Ga) plays a pivotal role in averting surface nanoparticle aggregation, and consequently, in preventing the formation of toxic *CH*_x_* and *CO species through the bridged adsorption of ethanol molecules. Contrastingly, when conventional Pt/C surfaces are employed, the cleavage of C─C bonds becomes a challenging task, further exacerbated by the laborious process of entirely oxidizing the small fraction of C1 species generated during the cleavage process, which ultimately leads to the deactivation of the catalytic active centers.

Wang et al. unveiled an elegant one‐pot synthesis technique for the fabrication of ternary PtCuRu nanoflowers.^[^ ^37^ ^]^ Novel nanostructures exhibit high‐index facets and display an enrichment of ruthenium (Ru) along their edges, strategically engineered to accelerate the kinetics of C─C bond cleavage. As depicted in Figure 2D, their findings reveal a marked reduction in the reaction barrier associated with the rate‐determining steps for EOR, involving the cleavage of the C─C bond, when conducted on the PtCuRu (111) surface in stark contrast to Pt (111). Further, during the EOR process, ruthenium (Ru) was introduced as an additional active center, significantly augmenting the adsorption state. The incorporation of copper (Cu) doping in this catalytic system effectively modulated the electronic structure of both platinum (Pt) and ruthenium (Ru), thereby expediting the reaction kinetics and facilitating the comprehensive oxidation of ethanol to CO_2_. In terms of stability, the activity of Pt_0.68_Cu_0.18_Ru_0.14_ NFs decreased by only 13% after 500 cycles. The result of analyzing the electrolyte using ICP‐MS indicates that there was no significant de‐alloying process. The TEM results after stability testing indicated no significant morphological changes.

**Figure 3:**
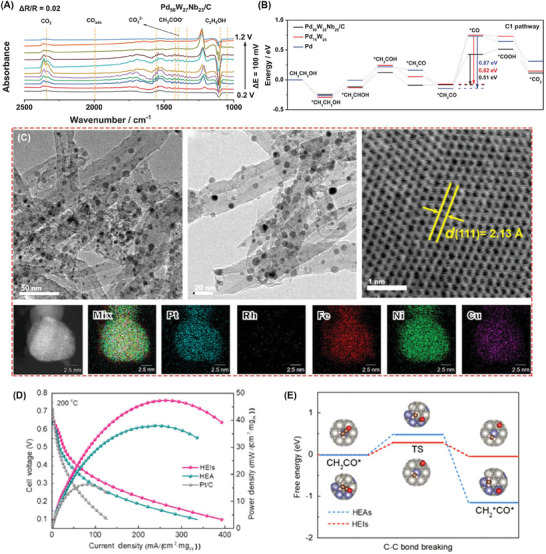
A) In situ FTIR spectra of ethanol oxidation reaction on the Pd_50_W_27_Nb_23_/C and B) DFT‐calculated reaction mechanisms of C1 pathway. Reproduced with permission.^[^ ^39^ ^]^ Copyright 2022, Wiley‐VCH. C) The morphology characterization of high‐entropy intermetallic.^[^ ^45^ ^]^ D) The high‐temperature DMFCs performance with different catalysts as anodes. E) The energy barriers of C─C bond breaking ordered and disordered alloy surfaces. Copyright 2022, Wiley‐VCH.

Similarly, the regulatory strategy of alloying extended to the design of Pd‐based electrocatalysts.^[^ ^38^ ^]^ Lai et al. found that niobium (Nb), owing to its oxophilic high‐valence characteristics when incorporated into a PdW alloy, assumed a pivotal role in facilitating C─C bond cleavage and the oxidation of CO intermediates.^[^ ^39^ ^]^ In situ FTIR spectroscopy (as depicted in **Figure** **3**A) reveals that the characteristic CO_3_^2−^ band at 1390 cm^−1^ exhibited higher intensity in Pd_50_W_27_Nb_23_/C than in the spectrum of Pd_97_W_3_/C. This heightened intensity suggested an elevated C1 selectivity conferred by the introduction of the niobium (Nb) site. Further, the peak at 2000 cm^−1^, associated with adsorbed *CO, gradually diminished with increasing applied potential, indicating accelerated CO oxidation on the Pd surface. In tandem with experimental results, density functional theory (DFT) calculations underscored the considerable reduction in energy barriers during the transition from CH_3_CO to CO on the PdWNb surface compared to pure PdW and Pd (Figure 3B). Liu et al designed quasi‐single‐crystalline mesoporous PdCu nanoplates at atomic dimension, with an excellent mass activity (6.09 A mg_Pd_^−1^) and C1‐product Faraday efficiency of 72.1%.^[^ ^40^ ^]^ The step position of Pd was a key center for adsorption and activation of EtOH, as well as Cu‐doping accelerating C─C bond cleavage and adsorbing OH groups, thereby further oxidizing *CO into CO_2_. Jin et al. achieved a scalable‐production strategy to prepare the size‐ and shape‐controllable amorphous PdCu nanomaterials, affording mass activity of 15.25 A mg_Pd_^−1^ and C1 path Faraday efficiency of 69.6%.^[^ ^41^ ^]^

High entropy alloys, characterized by their exceptionally heterogeneous elemental composition, present enticing avenues for elevating the efficacy of electrocatalytic systems.^[^ ^42^, ^43^, ^44^ ^]^ Drawing upon prior understanding of the discrete functional roles played by various constituent elements within the EOR process, Wang et al. have embarked on a pioneering endeavor to meticulously craft PtRhFeNiCu high entropy intermetallic compounds, strategically designated as anode catalysts for high‐temperature ethanol fuel cells.^[^ ^45^ ^]^ The morphology and lattice spacing of high entropy intermetallic compounds were characterized by TEM and high‐resolution STEM in Figure 3C. By harnessing the inherent precision within the atomic arrangement structure and harnessing lattice‐induced stress, the power density achieved within DEFCs outfitted with high entropy intermetallic alloys achieved a remarkable 47.5 mW cm^−2^ (as eloquently depicted in Figure 3D), surpassing that of conventional Pt/C catalysts by a notable factor of 2.17. Delving into the intricacies, the DFT analyses (Figure 3E) substantiate that the orchestrated atomic arrangement structure played an instrumental role in substantially abating the free energy barrier associated with the pivotal cleavage of C─C bonds within the CH_2_CO* intermediates. The EOR catalyst also demonstrated excellent stability for over 50 000 s operation.

**Figure 4:**
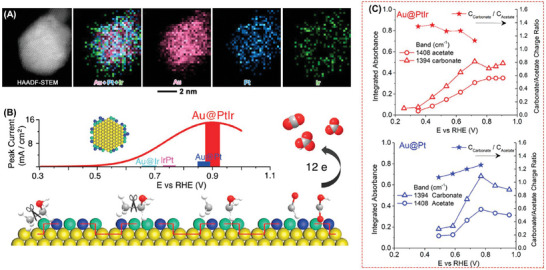
A) The HAADF‐STEM image and 2D mapping of Au@PtIr particle. B) Schematic diagram of the efficient mechanism of breaking C─C bonds by Au@PtIr. C) Integrated absorbances of EOR products and molar ratio of carbonate to acetate for Au@PtIr/C and Au@Pt/C. Reproduced with permission.^[^ ^50^ ^]^ Copyright 2019, American Chemical Society.

The synergistic amalgamation of a core–shell architectural framework with alloying dynamics demonstrates a highly efficacious strategy for fine‐tuning both electronic and lattice strain effects, thereby engendering a state of heightened catalytic efficiency conducive to the intricate electrochemical reactions.^[^ ^46^, ^47^, ^48^ ^]^ Yang et al reported Au@FePd, featuring sub‐nano FePd shells (**Figure** **4**A), displayed outstanding alkaline EOR performance (13.3 A mg_Pt_^−1^).^[^ ^49^ ^]^ Moreover, the electrolyte analysis revealed the presence of only 4.5 × 10^−4^ m acetic acid, a mere nineteenth of that detected with the Pd/C catalyst. As far as Au@PtIr is concerned, the Au‐induced tensile strain on PtIr surface facilitated C─C bond splitting via ethanol dissociative adsorption, and Ir promoted dehydrogenation at low potentials (Figure 4B), indicating that the synergistic effects of multiple mechanisms play a key role in improving the EOR performance for platinum‐based electrocatalysts.^[^ ^50^ ^]^ According to in situ FTIR spectra, the functions of absorption intensity and potential of intermediate products were summarized at Figure 4C for Au@PtIr and Au@Pt, respectively. The C_carbonate_/C_acetate_ was 1.3, approximately, for Au@PtIr/C from 0.3 and 0.6 V versus RHE, suggesting ≈57% of the current of the EOR process was generated via a direct 12‐electron pathway. While Au@Pt exhibited comparable selectivity, it is noteworthy that the initial potential of the detected product was significantly elevated when compared to Au@PtIr. This divergence could be attributed to the lattice strain generated by the presence of the Au core, which played a pivotal role in facilitating C─C bond cleavage. In contrast, the introduction of iridium (Ir) expedited the oxidative dehydrogenation of ethanol.

**Figure 5:**
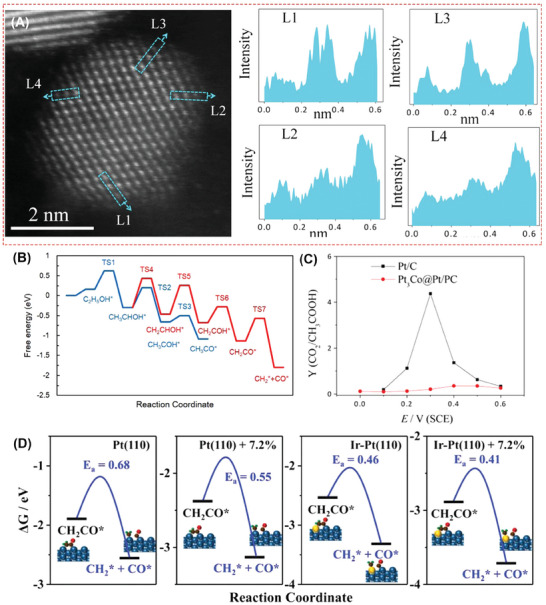
A) Atomic‐resolution ADF‐STEM image of Pt_3_Co@Pt/PC, with the intensity of sites L1, L2, L3, and L4. B) Free energy of EOR on the stepped Pt_3_Co (211) surface. C) Ratio change of integrated intensities of CO_2_ and intensities CH_3_COOH of Pt_3_Co@Pt/PC and Pt/C with different increasing overpotential. Reproduced with permission.^[^ ^56^ ^]^ Copyright 2017, American Chemical Society. D) The reaction barriers for breaking C─C in CH_2_CO* on different catalyst surfaces. Reproduced with permission.^[^ ^57^ ^]^ Copyright 2022, Wiley‐VCH.

Taking into account the operational parameters of catalysts within proton exchange membrane fuel cells, the high‐temperature, high‐humidity, and proton‐rich environment, it becomes imperative to incorporate the challenge of mitigating metal dissolution into the electrocatalyst design process.^[^ ^51^, ^52^ ^]^ Metal dissolution, if left unaddressed, will lead to irreversible proton channel blockage within the fuel cell system. The structure, platinum‐group‐metal shell and transition‐metal core, will not only produce a marked effect about regulating the d‐band of precious metals by base metals, but also decelerate dissolution phenomenon.^[^ ^53^, ^54^, ^55^ ^]^ Sun et al reported a convenient strategy for preparation of Pt_3_Co with Pt‐skin (**Figure** **5**A), only 1–2 atomic layers, ≈0.5 nm, to achieve efficient acid‐EOR process (0.79 mA µg_Pt_^−1^).^[^ ^56^ ^]^ According to DFT (Figure 5B), CH_2_CO* is the most suitable C2 intermediate produced by the EOR process for breaking the C─C bond on the stepped Pt_3_Co (211) surface, with an energy barrier of only 0.57 eV for forming CH_2_* and CO*. However, based on the integral intensity statistics of the characteristic peaks for C1/C2 intermediates in the in situ mass spectroscopy FTIR spectra (Figure 5C), the oxidation selectivity of Pt_3_Co@Pt/PC is lower than that of platinum carbon, indicating that creating complete oxidation sites proved in theoretical calculations still poses a challenge in real experimental synthesis. A heterogeneous hcp‐PtPb/fcc‐Pt core/shell hexagonal nanosheet with 7.2% tensile strain wrapped on the surface of Pt (110), with Ir single atom doping, was reported by Zhang et al. as an EOR electrocatalyst to achieve the C1 pathway selectivity up to 57.93%.^[^ ^57^ ^]^ As Figure 5D shows, the activation barriers of cleavage for C─C bond in CH_2_CO* intermediate on the surface of Pt(110), Pt(110)+7.2%, Ir‐Pt(110), and Ir‐Pt(110)+7.2% are 0.82, 0.75, 0.32, and 0.21 eV, respectively, which reflects the synergistic effect of tensile strain and Ir single atom doping on the ability of the electrocatalyst to break the C─C bond. In the above‐mentioned process, it is not difficult to find that Ir doping plays a more dominant function in reducing energy barriers and blocking the C2 pathway, prominently. The mass specific activity of PtPb@PtIr_1_ reaches 5.91 A mg_Pt+Ir_^−1^ and only loses 37.2% after 5000 cycles. The catalyst designs in the above work make EOR more inclined toward 12‐electron processes, which also means less CO and CH_3_COOH in the reaction system. Reducing toxic intermediates is the key to improving stability.

**Figure 6:**
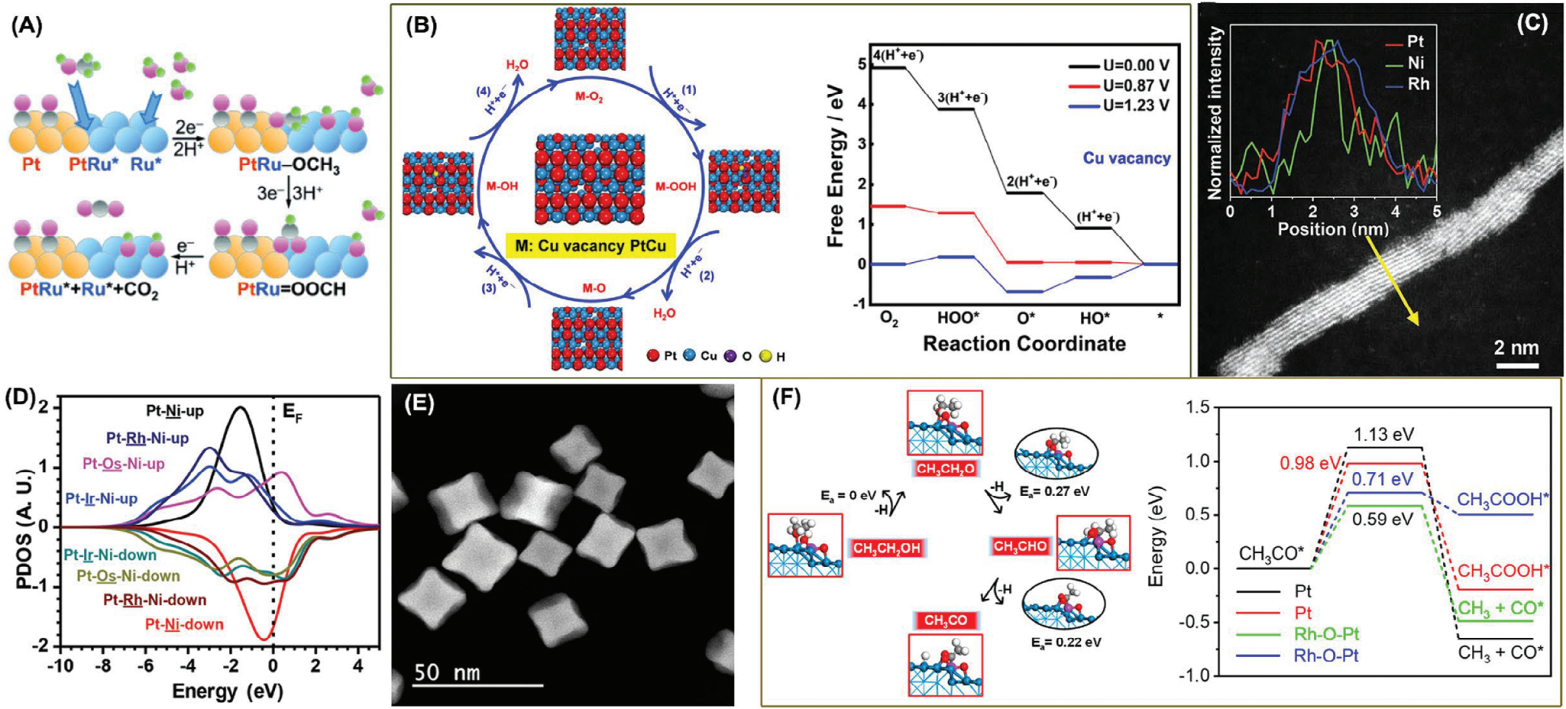
A) Oxidation mechanism of intermediate Co_ad_ with the assistance of OH* species. Reproduced with permission.^[^ ^65^ ^]^ Copyright 2015, Wiley‐VCH. B) Cu defected PtCu (111) surface and free energy diagrams on Cu defected PtCu (111) surface. Reproduced with permission.^[^ ^67^ ^]^ Copyright 2020, Wiley‐VCH. C) EDX line‐scanning profile of Pt_69_Ni_16_Rh_15_ nanowires. D) PDOSs of Ni 3d, Ir 5d, Os 5d, and Rh 4d bands. Reproduced with permission.^[^ ^32^ ^]^ Copyright 2019, Wiley‐VCH. E) HAADF‐STEM image of Rh–O–Pt dual sites. F) Scheme and computed energetics of ethanol being oxidized to the major product acetaldehyde and reaction energy barriers of CH_3_CO* oxidation and cleavage on Rh–O–Pt models. Reproduced with permission.^[^ ^71^ ^]^ Copyright 2023, American Chemical Society.

Actually, the secondary metal apart from Pt or Pd in the alloy can act as the secondary active site and forms a dual active site catalyst together with Pt or Pd, which embodies the concept of “1 + 1 > 2”.^[^ ^58^, ^59^ ^]^ Extensive investigations into alcohol oxidation have unequivocally substantiated the notion that the introduction of a secondary catalytic site yields an increased population of OH* species, thereby imparting essential impetus to the reaction kinetics, an imperative factor in the comprehensive oxidation of carbon–oxygen intermediates.^[^ ^60^, ^61^, ^62^ ^]^ Some oxophilic metals, such as Mo, Ru, and Rh have been used as assistant active sites to provide adsorbed OH* species, and then, to accelerate the oxidation of reaction intermediate *CO that strongly adsorbed on the Pt sites (**Figure** **6**A).^[^ ^63^, ^64^, ^65^, ^66^ ^]^ The introduction of assistant active sites can also weaken the adsorption of O* intermediate through enhancing the adsorption capacity of Pt atoms to the HO* intermediate.^[^ ^67^ ^]^ DFT calculations demonstrate that the O* + H^+^ + e^−^ → OH* step starts above 0.87 V with an overpotential as low as 0.36 V (Figure 6B). In addition, the electronic effect is invoked to explain the activity enhancement of EOR catalysts with dual sites.^[^ ^32^ ^]^ Guo et al. prepared ultrathin PtNi*M* (*M* = Rh, Os, and Ir) nanowires (Figure 6C) with excellent anti‐CO‐poisoning ability and high activity. Dynamic adsorption analysis confirms that the synergetic d‐orbital interplay between Pt and Rh/Ni can lower the Pt 5d band center, and then, promote the CO_ad_ intermediates toward full oxidization (Figure 6D). The easier desorption of *CO is also an important reason for the improved stability.

Hutchings,^[^ ^68^ ^]^ in their meticulous exploration of hydroxyl oxidation involving 5‐hydroxymethylfurfural (HMF) as a model reaction, convincingly demonstrated that the oxidation capability of discrete Pd and Au phases surpassed that of individual components, including alloys. In terms of the electro‐oxidation process of ethanol, Wang et al. made an insightful discovery: the catalytic activity of the Pd–Zn dual active site significantly outperformed that of the pure Pd–Pd site, achieving a remarkable enhancement, with a reaction activity 24‐fold superior to that of conventional Pd/C catalysts.^[^ ^69^ ^]^ In further research, Guo et al designed YO*_x_*/MoO*_x_*‐Pt NWs for the alcohol oxidation process, where YO*_x_*/MoO*_x_* was an important site for the oxidation of *CO intermediates.^[^ ^70^ ^]^ The coordination of dual sites in YO*_x_*/MoO*_x_* also accelerated the cleavage of C─C bonding, and the characteristic peak of CO_2_ would be observed at a low potential of ≈0.3 V in the infrared spectrum. Wang et al. constructed oxygen‐bridged long‐range Rh–O–Pt dual sites to accelerate C─C cleavage through redistributing the surface‐localized electron around Rh–O–Pt (Figure 6E).^[^ ^71^ ^]^ Theoretical calculations disclosed that the redistribution of the surface‐localized electron around Rh–O–Pt could lower the energy of C─C bond cleavage (0.59 eV, Figure 6F), accelerating C─C bond cleavage.

### 2.2 Cascade Electrocatalysis

The primary objective of the DEFCs is to convert ethanol into CO_2_ directly; yet, the C1 pathway shares a small part of the reaction due to the high bond dissociation energy for the C─C bond breaking (87.3 kcal mol^−1^) and the sluggish kinetic of C1‐pathway. Researchers spare no effort to optimize the design of the active site of the catalyst to improve C1 pathway selectivity. However, it is still difficult to achieve the complete oxidation of the ethanol and achieve the 100% selectivity of CO_2_. Ethylene is also a C2 type organic compound; while, its activation energy of C═C bond (6.4 kcal mol^−1^) is much lower than that of the C─C bond in ethanol. Thus, introducing ethylene as reaction intermediates may be an effective strategy to accelerate the C─C bond breaking kinetics and realize a higher C1‐pathway selectivity.

**Figure 7:**
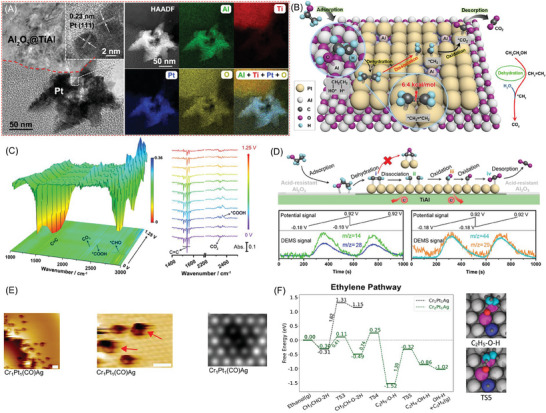
A) FIB‐HRTEM image, HAADF‐STEM image of Pt/Al_2_O_3_@TiAl and the corresponding elemental mapping. B) C1‐pathway, C) In situ FTIR spectra, and D) DEMS spectra and the corresponding reaction process of EOR on Pt/Al_2_O_3_@TiAl. Reproduced with permission.^[^ ^72^ ^]^ Copyright 2023, Wiley‐VCH. E) Zoomed‐out STM image of the CrPtAg alloy in the region around a step edge. F) Ethanol dehydrogenation to ethylene on Pt_1_Cr_1_Ag and Pt_1_Cr_2_Ag. Reproduced with permission.^[^ ^74^ ^]^ Copyright 2023, American Chemical Society.

On the basis, our group creatively develop a unique composite catalyst Pt/Al_2_O_3_@TiAl with cascade active sites to catalyze dehydration of ethanol and oxidation of ethylene, respectively (**Figure** **7**A).^[^ ^72^ ^]^ As a result, a C1‐pathway selectivity of 100 % during EOR is achieved via introducing ethylene as the precursor for the C─C bond breaking (Figure 7B). On Pt/Al_2_O_3_@TiAl, ethanol is first dehydrated on the Al_2_O_3_@TiAl support to form ethylene owing to the dehydration of Al_2_O_3_ to ethanol. The dehydration occurs through an elimination mechanism, in which the β‐hydrogen of the ethanol is transferred to the surface oxygen of the oxides and the C─OH bond is depleted in a concerted reaction step.^[^ ^73^ ^]^ In addition, the formed ethylene is further oxidized on Pt catalyst to CO_2_ through cleaving the C═C bond, which is confirmed by the in situ Fourier transform infrared spectroscopy (Figure 7C) and differential electrochemical mass spectrometry (Figure 7D) analysis. As a result, this unique catalyst shows outstanding EOR performance (3.83 mA cm^−2^_Pt_) and high stability with only 7% current loss after 12 h electrochemical test.

Coincidentally, Montemore et al. discovered that the dehydration of ethanol could occur on Pt–Cr sites in PtCrAg (Figure 7E) and produce ethylene when the dopant concentrations of PtCr ensembles was high (Figure 7F).^[^ ^74^ ^]^ The selectivity to ethylene could reach to ≈80% at 10% dopant concentrations, which provided the conditions for ethylene‐mediated cascade electrocatalysis for complete oxidation of ethanol. This unique pathway of ethanol first dehydrating on the first active sites, and then, further oxidizing on the second active site could provide guidance to the further study of complete ethanol electro‐oxidation; we expect more cascade catalytic pathways to be found to realize the full utilization of ethanol.

### 2.3 Interface Synergism

**Figure 8:**
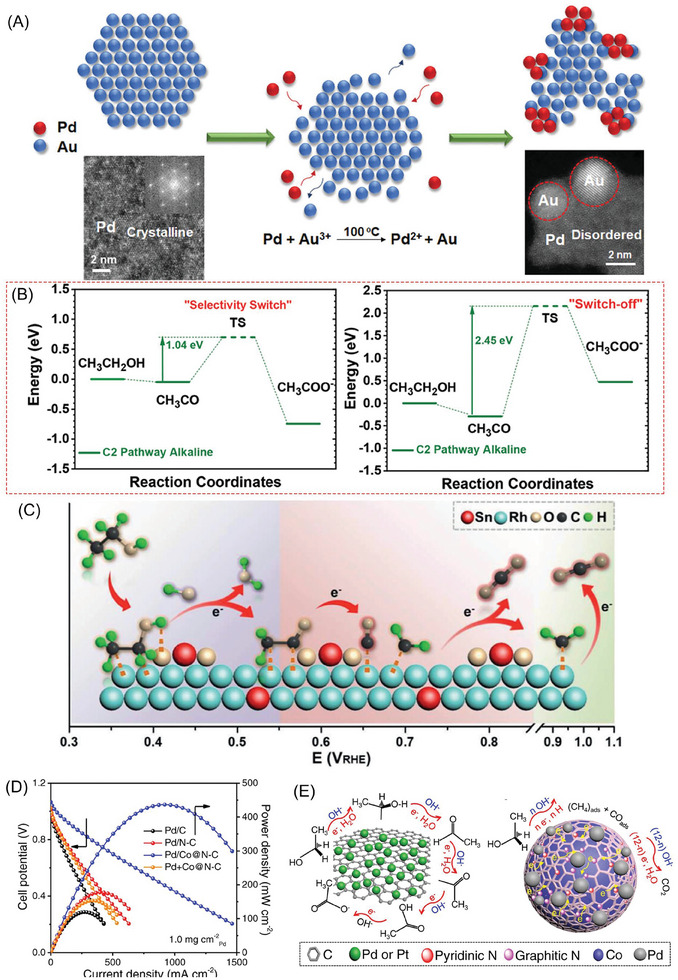
A) The schematic diagram of synthesis path for 2D Pd–Au heterophase nanosheet. Reproduced with permission.^[^ ^30^ ^]^ Copyright 2021, Wiley‐VCH. B) Comparison of oxidation energy barriers for CH_3_CO species on different catalyst surfaces. C) Schematic diagram of accelerating C─C bond clearance on Rh–SnO_2_ interface. Reproduced with permission.^[^ ^17^ ^]^ Copyright 2021, Wiley‐VCH. D) The polarization curves and power density for different Pd‐based electrocatalyst. Reproduced with permission.^[^ ^86^ ^]^ Copyright 2023, Springer Nature Ltd. E) Schematic diagram of EOR process comparison between Pd/Co@N–C and Pd/C.

Given the intricacies inherent to the EOR process, it is often imperative to employ multi‐component catalyst systems, where the synergistic collaboration among diverse constituents becomes a prerequisite for achieving comprehensive oxidation.^[^ ^11^, ^75^ ^]^ The interface synergy between different components assumes paramount research significance as it represents a pivotal determinant for facilitating the elusive 12‐electron process. For example, 2D Pd‐Au heterophase nanosheet (Pd‐Au HNS),^[^ ^30^ ^]^ with abundant interphase between amorphous Pd domain and crystalline Au cluster, is prepared through a simple galvanic reaction (**Figure** **8**A). In the displacement reaction, chloroauricate ion reacts with the low‐coordination Pd atom at the edge site, disrupting the ordered single‐crystal structure and forming a unique interface. Among the series of catalysts with different ratios of Pd and Au, the Pd–Au HNS, with atom ratio as 88:12, exhibits the best mass specific activity of 9.1 A mg_Pd_^−1^_,_ which is 9.3 and 7 times higher than traditional palladium carbon and platinum carbon, respectively. What's even more interesting is that the novel interface design, as a switch‐off, to some extent, blocks the C2 pathway. Compared to the surface of Pd NS, the activation energy for further oxidation of CH_3_CO intermediate to acetic acid on Pd–Au HNS increases from 1.04 to 2.45 eV (Figure 8B), notably, which has a critical impact on the 33.2% C1 pathway selectivity exhibited by a simple‐structure catalyst.

Rh is more susceptible to poisoning and deactivation by carbon oxygen and hydrocarbon intermediates in the EOR process, making it rare for rhodium‐based electrocatalyst to serve as active center.^[^ ^76^ ^]^ Metal‐oxide catalyst always play a crucial role in various complex oxidation reactions.^[^ ^77^, ^78^, ^79^ ^]^ Huang et al.^[^ ^17^ ^]^ demonstrated that constructing Rh–SnO_2_ active interface (Figure 8C) will efficiently achieve a 12‐electron EOR process, with a Faraday efficiency of up to 72.8%. Due to the presence of the interface, the electrons of Rh transfer to SnO_2_, causing its *d*‐band to shift upward, which accelerates the cleavage of C─C bonds and the desorption process of C1 intermediates. Through CO‐DRIFTS, it will be observed that the interface formed by the addition of SnO_2_ significantly weakens the peak strength of carbonyl edge adsorption and CO bridge adsorption, accompanied by a blue shift of the peak (from 1838 to 1860 cm^−1^). In addition, the strong interaction between Rh and Sn species at the Rh─SnO_2_ interface significantly boosts the EOR performance in terms of high stability.

Palladium, with its akin electronic structure to platinum, has garnered recognition as a plausible alternative in both anodic and cathodic reactions within the domain of fuel cells.^[^ ^80^, ^81^, ^82^ ^]^ Nonetheless, it is pertinent to acknowledge that palladium exhibits a heightened affinity toward diverse oxygen species, a characteristic that significantly constrains its utility within the realm of electrocatalysis.^[^ ^83^ ^]^ During EOR, it is noteworthy that the selectivity pertaining to the 12‐electron process on the Pd surface registers at a mere range of 0.5–7.5%.^[^ ^84^, ^85^ ^]^ Based on the previous understanding of Pd electrocatalytic performance, Yang et al.^[^ ^86^ ^]^ proposed an interface synergy strategy, involving the electron transfer occurring at the interface between Pd and Co@N–C, thereby reducing the electron cloud density on the surface of Pd. Interestingly, through the simultaneous activation of ORR and EOR processes by unique interface design, the power density of anion exchange membrane fuel cells reached 438 mW cm^−2^ with 1 mg_Pd_ cm^−2^ (Figure 8D) and exhibited stability over 1000 h at 0.5 V. Further, at a low potential of 0.2 V, Pd/Co@N–C would promote C─C bonding cleavage, as well as, the C1 intermediate would be further oxidized at 0.7 V versus RHE, resulting in an exceptional 12‐electron process (Figure 8E). However, traditional Pd/C, even when physically mixed with Co@N–C, is still a general four‐electron process, demonstrating the irreplaceable role of the interface effect.

### 2.4 Doping Effect

Regarding the fact that heterogeneous catalytic reactions mostly occur on the surface/interface between the catalysts and reactants, surface active sites play vital roles in the catalytic reactions. Inspired by the preparation of single‐atoms catalysts (SACs), single‐atom alloy (SAA) catalysts have already been developed.^[^ ^87^ ^]^ Such methods have received extensive attention in catalysis due to their unique reaction sites and high reactivity; however, few of such catalysts show satisfying C_1_ pathway selectivity in practical application, especially in the acidic solution.^[^ ^88^ ^]^ On the basis, researchers find that doping foreign atoms to construct surface defect can be a promising method to promote the performance of heterogeneous catalysts in the electrocatalysis process.^[^ ^89^ ^]^ The doping to Pt electrodes by other metals such as Ru, Rh, Sn, Pd has been investigated to be effective in increasing the peak current density and lowering the onset potential significantly;^[^ ^90^, ^91^, ^92^, ^93^, ^94^ ^]^ while, most of the main products are acetic acid and acetaldehyde. Thus, it is of vital importance to give consideration to both raising the full utilization of ethanol and enhancing the catalytic activity.

**Figure 9:**
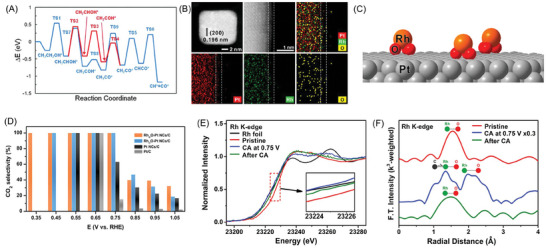
A) Energy profiles of ethanol electrooxidation on Pt(111). The blue line is the energy profile of the α‐dehydrogenation pathway and the red line is that of the β‐dehydrogenation pathway. Reproduced with permission.^[^ ^95^ ^]^ Copyright 2014, Royal Society of Chemistry. B) High‐angle annular dark‐field STEM images, EDS mapping image, and C) schematic model of Rh_at_O–Pt NCs. D) The CO_2_ selectivity of Rh_at_O–Pt NCs and other samples from 0.25 to 1.05 V. E) XANES and F) EXAFS spectra for the Rh K‐edge of Rh_at_–O Pt NCs/C before, after, and during EOR chronoamperometric test in 0.1 m HClO_4_ + 1.0 m ethanol solution at 0.75 V. Reproduced with permission.^[^ ^97^ ^]^ Copyright 2022, Washington, DC.

In 2014, Lin group^[^ ^95^ ^]^ found that CH_3_CHOH* is the key intermediate during ethanol electrooxidation and the activity of β‐dehydrogenation is the rate determining factor that affects the CO_2_ selectivity of ethanol electro‐oxidation using the first principles method (**Figure** **9**A). They demonstrated that the doping of some transition metals such as Ru, Os, Rh, and Ir can accelerate b‐dehydrogenation as their doping can decrease the barrier from CH_3_CHOH* to CH_2_CHOH*. Besides, the formation of *OH by the water dissociation would lead to the formation of acetaldehyde and acetic; therefore, the doping of Ru, Os, and Ir atoms would lead to the non‐CO_2_ pathway as they may decrease the formation potential of *OH. Similarly, in 2016, Michel group^[^ ^96^ ^]^ reported that though Pt(100) surface shows higher ethanol to CO_2_ conversion compared to Pt(111) and Pt(110), it prefers partial oxidation to form acetic acid (CH_3_COOH) owing to the impeded kinetics of the C─C bond cleavage. They also suggested that destabilizing the gem‐diol, stabilizing the acetyl, and facilitating its splitting to CH_3_ and CO may contribute to a higher CO_2_ selectivity.

**Figure 10:**
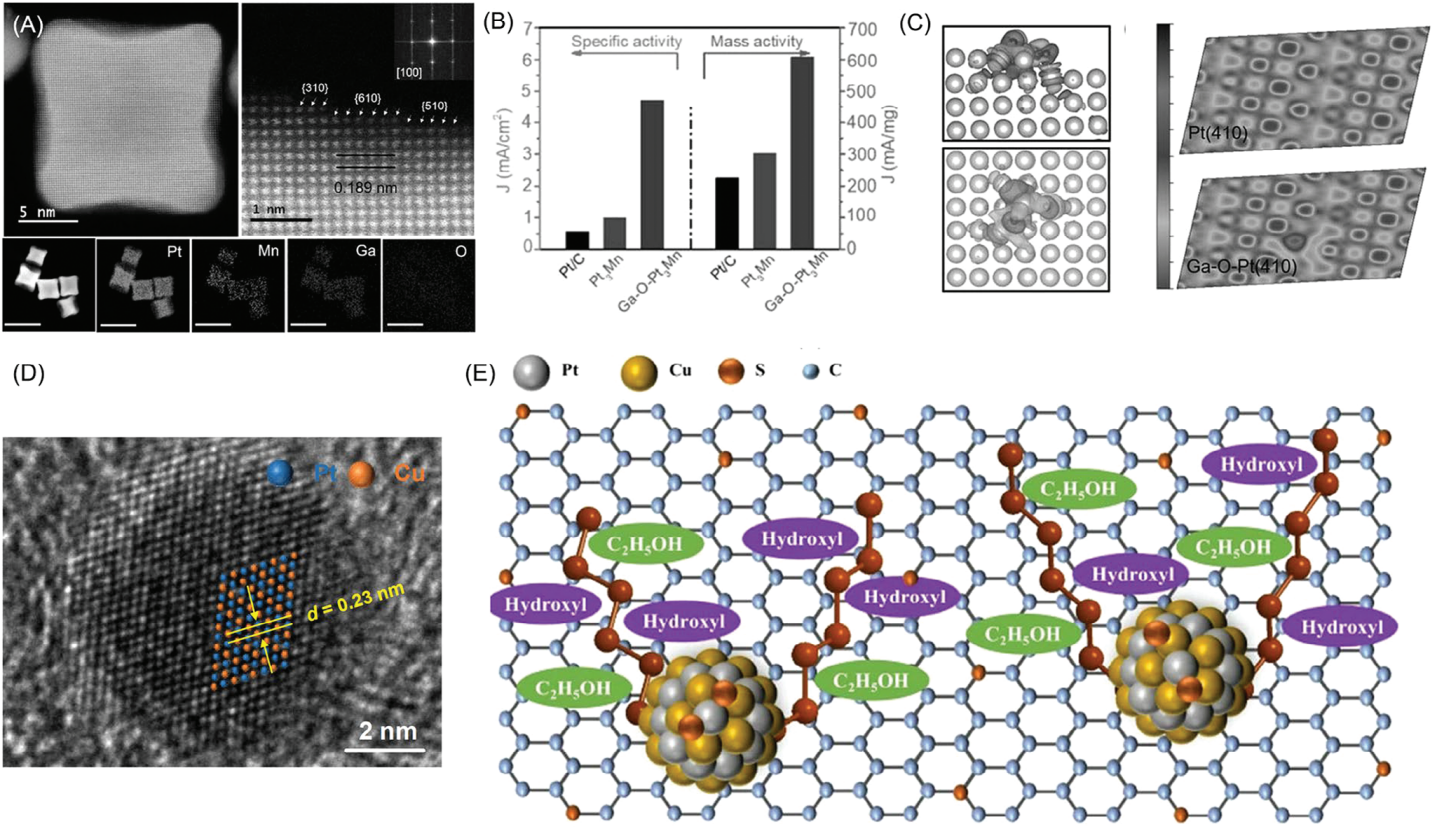
A) Aberration‐corrected HAADF‐STEM image and STEM‐EDS elemental mapping of the Ga–O–Pt_3_Mn nanocatalyst. B) The specific activities and mass activities of Ga–O–Pt_3_Mn and other samples. C) The charge density difference of Ga–O–Pt(410) interface and the electron localization function analysis mapped for Pt(410) and Ga–O–Pt(410) structure. Reproduced with permission.^[^ ^88^ ^]^ Copyright 2022, Wiley‐VCH. D) HRTEM image of S_3.67_‐PtCu intermetallic/C. E) Enriching effect enhances cleavage of C─C bond. Reproduced with permission.^[^ ^98^ ^]^ Copyright 2023, Springer.

Chen group^[^ ^97^ ^]^ succeeded in the controlled synthesis of dispersing partially oxidized single Rh on the (100) surface of Pt nanocubes (Rh_at_O–Pt NCs; Figure 9B,C), which raised CO_2_ selectivity to above 99.9% from 0.35 to 0.75 V (Figure 9D). The doping of Rh atoms helped to break C─C bond and remove the poisoning *CO; thus, decreasing the CO_2_ generation potential to be much closer to the thermodynamic potential for the oxidation of ethanol to CO_2_ (0.143 V) than the commercial Pt/C. DFT calculations have found that the adsorption of CO on Rh_at_O/Pt (100) sites is significantly weaker than on Pt (100) site. The in situ XAFS analysis conducted at the Rh K‐edge using the chronoamperometric mode of EOR at 0.75 V suggested a unique environment of Rh_at_O on the Pt(100) surfaces as a new active site, which contributed to the astonishing CO_2_ selectivity (Figure 9E,F). Xu group^[^ ^87^ ^]^ used monodispersed metal sites to tailor Pt‐based nano‐catalysts and first construct monodispersed Ga on Pt_3_Mn nanocrystals (Ga–O–Pt_3_Mn, **Figure** **10**A) with high‐indexed facets. The specific activity of the reported Ga–O–Pt_3_Mn nano‐catalyst was 3.68 and 8.41 times that of Pt_3_Mn and commercial Pt/C (Figure 10B). The theoretical modeling results confirm a strong electronic interaction between the Pt substrate and the monodispersed Ga component via the unconventional p–d orbital hybridization. The doping of Ga monodispersed atoms upshifted d‐band center from the Fermi level, which could strengthen the adsorption of ethanol molecules (Figure 10C).

In addition to the above strategies, tailoring amorphous electrocatalysts with nanostructure and modifying electrocatalysts with polysulfide can also facilitate the C─C bond cleavage significantly. Jin et al. utilized amorphous nanocatalysts with the enriched coordination unsaturated surface sites to cleave the chemically stable C─C bond.^[^ ^41^ ^]^ Their DFT calculations results revealed that the energy barrier C─C bond breaking on coordination of unsaturated surface sites (0.29 eV) was much lower than on the crystalline surface (0.85 eV). As a result, the amorphization of PdCu catalysts afforded a C1 path Faraday efficiency as high as 69.6%. To accelerate the C─C bond cleavage, Wang et al. employed sulfur bridge structure and polysulfide bond to enrich hydroxyl and ethanol for PtCu intermetallic (Figure 10D).^[^ ^98^ ^]^ The enrichment effect of ethanol and hydroxyl on the catalyst surface could accelerate the oxidation of CO and expose a large number of active sites for splitting the C─C bond and improving the CO_2_ selectivity of EOR (Figure 10E). The CO_2_ selectivity of PtCu intermetallic nanocatalyst without polysulfide reached 93.5%.

## 3 Summary and Outlook

Direct ethanol fuel cell is an emerging power device that has demonstrated unique advantages in volumetric energy density (6.28 kWh·L^−1^), energy conversion efficiency, recharging time (3 min), and environmental protection.^[^ ^99^ ^]^ At present, electrocatalysts for oxygen reduction reaction at the cathode and proton exchange membrane have made great progress. Nevertheless, the activity and stability of commercial catalysts for ethanol oxidation reaction need to be further improved to meet the scale application requirements of DEFCs. We think that the electrocatalysts for ethanol oxidation should have the following characteristics: 1) Outstanding capacity to cleave C─C bond. Compared with C1 pathway (complete oxidation of ethanol with 12 electrons transferring), the C2 pathway only involves four electrons transferring without breaking a C─C bond during incomplete oxidation of ethanol, which seriously hinders the energy density output of DEFCs. However, the C─C bond activation is the rate‐limiting step for ethanol complete oxidation owing to the high activation energy of the C─C bond (87.3 kcal mol^−1^). Taking advantage of the different adsorption strength of the two carbon atoms via bridge‐type adsorption of ethanol molecules on heterogeneous dual active sites and constructing electrocatalysts with specific structure to achieve complete oxidation of ethanol by relay catalysis are directions worth focusing on in the future. 2) Remarkable stability. In most cases, the agglomeration and Ostwald's ripening of Pt nanoparticles are the main reasons for the degradation of catalyst.^[^ ^100^, ^101^, ^102^ ^]^ Developing corrosion resistant supports and enhancing the interaction between the active sites and supports have shown substantial promise up to now. 3) Acceptable cost. Extensive use of precious metals in catalyst layer is a main reason for the high cost of DEFCs. Optimizing the catalyst layer, such as constructing porous support to improve the utilization of precious metals and reduce the loading thereof, may be effective in reducing costs.

## Conflict of Interest

The authors declare no conflict of interest.
